# Platform enabling population-level imaging and data linkage in Scotland

**DOI:** 10.23889/ijpds.v9i5.2906

**Published:** 2024-09-18

**Authors:** James Sutherland, Thomas Nind, Ruairidh MacLeod, Andrew Brooks, Bianca Prodan, Susan Krueger, Emily Jefferson

**Affiliations:** 1 University of Dundee; 2 University of Edinburgh

Scotland has more than five million people within a single health system, using a single central Picture Archiving and Commication System (PACS) for radiography data. This enabled the project team to build a research resource exceeding a petabyte of imaging from 2010 onwards, with open-source tooling to collate and de-identify images on demand. Image metadata, treatment and diagnostic records can be used to define large cohorts of patients then make the data available remotely to researchers in a Trusted Research Environment (TRE).

Original identifiable images are stored in one of three isolated zones with controlled data transfer to protect patient privacy and prevent inadvertent disclosure. Linkage to other records is performed in the second zone on de-identified images using encrypted patient identifiers. Automated screening with optical character recognition and natural language processing was implemented to identify and redact personally identifiable information before release to researchers in the third zone.

A recent extension to this system has provided an ongoing feed of routine imaging, which is securely shared with regional counterparts to ensure the minimal possible additional load is placed on clinical PACS resources and avoid duplicate requests.

The project launched in April 2022 and since then a variety of research projects have already used this environment and data representing millions of pounds of funding, some using large cohorts with historical data up to 14 years old, re-assessing historical scans with the benefit of subsequent diagnosis to investigate possible early warning signs of conditions including dementia and pre-cancerous lung nodules.

